# Cancer in Families

**DOI:** 10.1371/journal.pmed.0010069

**Published:** 2004-12-28

The Icelandic population is now a part of a unique epidemiological study, which has involved investigating the genetic heritage of many of them. The reason that this experiment can be done is because of the remarkable records that exist in Iceland. Not only is there almost complete genealogical information dating back to the 18th century on all current (288,000) and many previous Icelanders (more than 600,000 in total), but in addition the country has an almost complete cancer registry dating from 1955. A company, deCODE Genetics, was set up to mine health-care data in Iceland, and to use it to assess the effect of genetics on health. Initially, the company attracted criticism, with some questioning the ethics of providing access to health-care data for many disease projects to a for-profit company. But the company has been supported by many Icelanders themselves, demonstrated by Icelanders donating blood samples with informed consent for research on multiple diseases, and now the project's scientific value is becoming apparent.

One such analysis is the subject of a paper by Laufey Amundadottir and colleagues in this month's *PLoS Medicine* that assesses how much genetic factors contribute to cancer risk across the whole Icelandic population.

The paper looked at 27 different types of cancers (all those with more than 200 cases) that had been registered between 1955 and 2002 and analysed the frequency of close and distant relatives also having that cancer, or another kind of cancer. Of the 27 cancers, 16 showed significant “familiality,” and for some this risk even extended to distant (that is, third- to fifth-degree) relatives. The seven cancers with the highest increased familial occurrence both in close and distant relatives were breast, prostate, stomach, lung, colon, kidney, and bladder cancers. And, interestingly, three cancers—stomach, lung, and colon cancer—were also seen more frequently in mates of patients, indicating a shared environmental risk factor. And for some cancers there was a familial association with other cancers, for example, relatives of individuals with stomach, colon, rectal, or endometrial cancer were more likely to have any of these cancers.[Fig pmed-0010069-g001]

**Figure pmed-0010069-g001:**
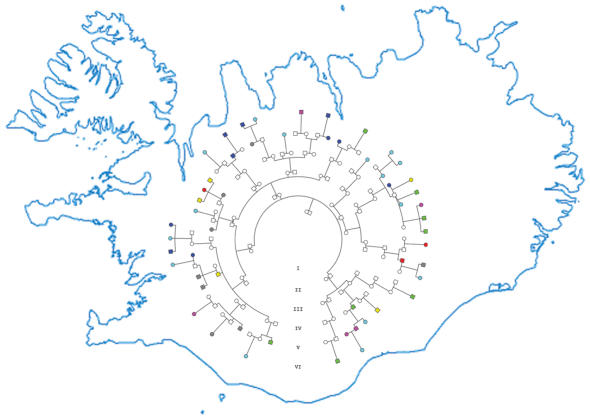
Icelandic genetics and genealogy

Cathryn Lewis, the academic editor for the paper comments on the study's strengths. “This level of family relationship and clinical diagnosis is rarely available from interviewing patients and family members. The size of the study (over 600,000 individuals, with 32,000 cancer cases) and the high quality of data enables the authors to detect subtle effects across distant relationships.”

How robust are these data, and what do they mean for the biological understanding of cancer? As Lewis says, “Although the current study is impressive in its size and scope, even here, the sample size becomes an issue, with the most convincing results seen in the most common cancers.” Certainly not all the findings are surprising; some rare cancers are already known to be associated with particular genetic defects, and syndromes that predispose to multiple cancers have been described, for example, that of Hereditary Nonpolyposis Colorectal Cancer. Other associations are more intriguing—the cluster of related cancers that include prostate, kidney, and bladder could possibly have a developmental origin, since all arise from the same part of the embryo.

So, by highlighting these subtle links, the study's particular value may become apparent: deciding future avenues of investigation in the complex interrelationships that interact to produce cancer.

